# A Multivariate Diagnostic Model Based on Urinary EpCAM-CD9-Positive Extracellular Vesicles for Prostate Cancer Diagnosis

**DOI:** 10.3389/fonc.2021.777684

**Published:** 2021-11-24

**Authors:** Yibei Dai, Yiyun Wang, Ying Cao, Pan Yu, Lingyu Zhang, Zhenping Liu, Ying Ping, Danhua Wang, Gong Zhang, Yiwen Sang, Xuchu Wang, Zhihua Tao

**Affiliations:** ^1^ Department of Laboratory Medicine, The Second Affiliated Hospital of Zhejiang University School of Medicine, Hangzhou, China; ^2^ Zhejiang University School of Medicine, Hangzhou, China; ^3^ Department of Laboratory Medicine, The First People’s Hospital of Yuhang District, Hangzhou, China

**Keywords:** extracellular vesicle, EpCAM, chemiluminescent immunoassay, prostate cancer, multivariate diagnostic model

## Abstract

**Introduction:**

Prostate cancer (PCa) is one of the most frequently diagnosed cancers and the leading cause of cancer death in males worldwide. Although prostate-specific antigen (PSA) screening has considerably improved the detection of PCa, it has also led to a dramatic increase in overdiagnosing indolent disease due to its low specificity. This study aimed to develop and validate a multivariate diagnostic model based on the urinary epithelial cell adhesion molecule (EpCAM)-CD9–positive extracellular vesicles (EVs) (uEV_EpCAM-CD9_) to improve the diagnosis of PCa.

**Methods:**

We investigated the performance of uEV_EpCAM-CD9_ from urine samples of 193 participants (112 PCa patients, 55 benign prostatic hyperplasia patients, and 26 healthy donors) to diagnose PCa using our laboratory-developed chemiluminescent immunoassay. We applied machine learning to training sets and subsequently evaluated the multivariate diagnostic model based on uEV_EpCAM-CD9_ in validation sets.

**Results:**

Results showed that uEV_EpCAM-CD9_ was able to distinguish PCa from controls, and a significant decrease of uEV_EpCAM-CD9_ was observed after prostatectomy. We further used a training set (N = 116) and constructed an exclusive multivariate diagnostic model based on uEV_EpCAM-CD9_, PSA, and other clinical parameters, which showed an enhanced diagnostic sensitivity and specificity and performed excellently to diagnose PCa [area under the curve (AUC) = 0.952, P < 0.0001]. When applied to a validation test (N = 77), the model achieved an AUC of 0.947 (P < 0.0001). Moreover, this diagnostic model also exhibited a superior diagnostic performance (AUC = 0.917, P < 0.0001) over PSA (AUC = 0.712, P = 0.0018) at the PSA gray zone.

**Conclusions:**

The multivariate model based on uEV_EpCAM-CD9_ achieved a notable diagnostic performance to diagnose PCa. In the future, this model may potentially be used to better select patients for prostate transrectal ultrasound (TRUS) biopsy.

## Introduction

Prostate cancer (PCa) is one of the most frequently diagnosed cancers and the leading cause of cancer death in males worldwide (1). Despite the widespread use of prostate-specific antigen (PSA) as a noninvasive screening tool for PCa, the low specificity of PSA has led to an increase in either overdiagnosis or unnecessary biopsies, especially when its value is within the PSA gray zone (4–10 ng/ml) (2, 3). Thus, it is urgently needed to explore new biomarkers for more accurate PCa diagnosis.

Urine is an ideal source of PCa biomarkers because the samples can be collected noninvasively in large amounts, and several urinary markers have been reported such as prostate cancer antigen-3 *(PCA3)*, transmembrane protease serine-2 *(TMPRSS2)*, and glutathione S-transferase P *(GSTP1)* gene (4–7). Recently, urinary extracellular vesicles (uEVs) have sparked interest as potential biomarkers (8, 9). uEVs are low-density membrane vesicles containing lipids, proteins, DNA, mRNAs, and microRNAs (10). A reproducible method for uEV isolation has been described by Pisitkun et al. (11) in 2004 and has been widely adopted for uEV analysis. Previous proteomic analysis of uEVs has revealed varieties of cancer-specific proteins in their cargoes (12, 13). However, the question remained whether there is a specific protein in uEVs that could provide diagnostic information for PCa and also be easily detected.

Epithelial cell adhesion molecule (EpCAM) is a transmembrane glycoprotein that plays an important role in Ca2+-independent hemophilic cell-to-cell adhesion, cell signaling, migration, proliferation, and differentiation of cancer cells (14, 15). It has thus gained considerable attraction as an appealing candidate biomarker for cancer diagnosis due to its strong expression in various carcinomas and their metastases compared with normal epithelia (16, 17). Recently, EpCAM on tumor-derived EV membrane was also employed as a promising tumor surface marker, while the tetraspanin family of proteins, such as CD63, CD9, and CD81, was mainly used as EV universal markers (18, 19). The use of these biomarkers to identify EVs from bodily fluids has garnered much interest as a non-invasive liquid biopsy for cancer.

**Figure 1:**
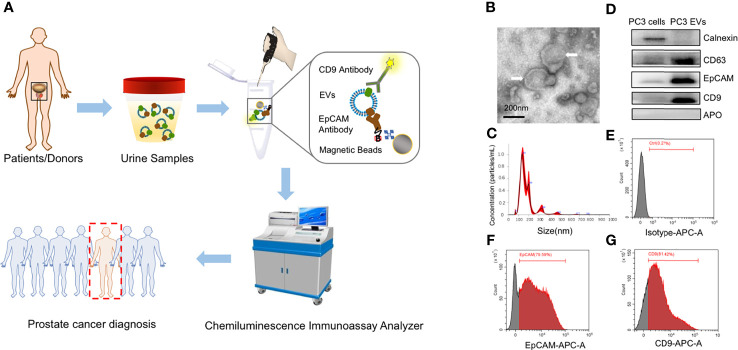
The scheme of workflow for urinary EpCAM-CD9-positive extracellular vesicle (uEV_EpCAM-CD9_) detection. **(A)** EpCAM-CD9-positive EVs diffused in urine are bound with acridinium ester (ACE)-labeled anti-CD9 antibodies and captured by magnetic microbeads labeled anti-EpCAM antibodies. After incubation for 60 min, uEV_EpCAM-CD9_ binding with magnetic microbeads can be easily isolated under an external magnetic field and quantitatively analyzed by a chemiluminescent immunoassay analyzer to diagnose prostate cancer. **(B)** TEM images of EVs isolated by ultracentrifugation (white arrow). **(C)** EVs are characterized by NTA. **(D)** The expression of CD63, CD9, EpCAM, calnexin, and APO in PC3 cell lysates and the EV fraction from PC3 by WB analysis. **(E–G)** Flow cytometry assay identified that approximately 80% of EVs released by PC3 carried EpCAM and CD9. EpCAM, epithelial cell adhesion molecule; uEVEpCAM-CD9, urinary EpCAM-CD9-positive extracellular vesicles; EVs, extracellular vesicles; ACE, acridinium ester; TEM, transmission electron microscope; NTA, nanoparticle tracking analysis; WB, western blot.

Accordingly, we herein aimed to develop and validate a multivariate diagnostic model based on the urinary EpCAM-CD9-positive EVs (uEV_EpCAM-CD9_) to improve the diagnosis of PCa. We first investigated the performance of uEV_EpCAM-CD9_ for the diagnosis of PCa using a newly laboratory-developed chemiluminescent immunoassay (CLIA) (**Figure 1A**). Briefly, uEV_EpCAM-CD9_ diffused in urine is bound with acridinium ester (ACE)-labeled anti-CD9 antibodies and captured by magnetic bead-labeled anti-EpCAM antibodies, followed by a thorough isolation under an external magnetic field. Consequently, the concentrations of EpCAM-CD9-positive EVs (EV_EpCAM-CD9_) can be quantitatively determined by measuring the chemiluminescent signals. Results indicated that EV_EpCAM-CD9_ from the culture supernatant of PCa cell lines were significantly elevated under the simulated tumor microenvironment. Moreover, preliminary results showed that uEV_EpCAM-CD9_ could distinguish patients with PCa from control sets, indicating that uEV_EpCAM-CD9_ may be a potential biomarker for PCa diagnosis. We then applied machine learning to training sets and subsequently evaluated the multivariate diagnostic model based on uEV_EpCAM-CD9_ in validation sets.

## Materials and Methods

### Cell Lines and Culture

Two human PCa cell lines (PC3 and LNCaP) and an immortalized prostate epithelial cell line (RWPE-1) were obtained from the American Type Culture Collection (Manassas, VA, USA). All cell lines were cultured in RPMI 1640 medium (Gibco Invitrogen, Carlsbad, CA, USA) supplemented with 10% fetal bovine serum (FBS; Thermo Fisher Scientific, MA, USA), 100 U/ml penicillin, and 100 µg/ml streptomycin in an incubator with 5% CO_2_ at 37°C.

### Urine Collection

Urine samples from 193 participants [112 PCa patients, 55 benign prostatic hyperplasia (BPH) patients, and 26 healthy donors (HDs)] were collected in the Second Affiliated Hospital of Zhejiang University School of Medicine. Approval was obtained from the Second Affiliated Hospital of Zhejiang University School of Medicine Ethical Committee before initiating the study. Detailed information on the patients is summarized in **Supplementary Table S1**. All methods were performed in accordance with the relevant guidelines and regulations. All the patients met the following inclusion criteria: (1) undergoing prostate biopsy for the first time, (2) three-dimensional size of the prostate available *via* transabdominal ultrasonography before biopsy, (3) blood tests performed within 1 week before biopsy, (4) complete clinical and pathological data available, (5) absence of acute prostatitis or systemic inflammatory disease, (6) absence of urinary tract infection, (7) no history of prostate surgery, (8) no history of 5-alpha reductase inhibitor use, and (9) no anti-inflammatory drug use within 2 weeks before blood tests. Initial voided urine (5–10 ml) was prospectively collected from patients at the time of day most convenient to the person before prostate biopsy. Matched urine samples were collected from PCa patients prior to (n = 10) and a week after local treatment by radical prostatectomy (n = 10).

### Extracellular Vesicle Isolation From Cell Culture Medium and Urine

EVs were isolated from cell culture medium by ultracentrifugation as previously described (20). Briefly, when 70%–80% confluency was reached, cells were washed twice with phosphate-buffered saline (PBS; pH7.0) and then incubated for 48 h in FBS-free medium. Cell culture medium was collected and subjected to consecutive centrifugation steps (300 × g for 10 min and 2,000 × g for 20 min) to remove dead cells and cellular debris. The supernatant was vacuum filtered using a 10-kDa centrifugal filter (Merck Millipore, Darmstadt, Germany), and EV concentrates were ultracentrifuged at 100,000 × g for 70 min at 4°C (Type 70 Ti Fixed-angle Titanium Rotor, k factor = 157.4) (Optima™ XP ultracentrifuge; Beckman Coulter, Indianapolis, IN, USA). Pellets were washed with PBS followed by ultracentrifugation at the same speed and time. The supernatant was discarded, and the resulting EV pellets were suspended in PBS and stored at -80°C.

In order to obtain uEVs, urine samples from patients with PCa, BPH and HDs were centrifuged at 3,000 × g for 20 min at 4°C to remove debris and then ultracentrifuged at 200,000 × g for 2 h at 4°C. The supernatant was removed, and the uEV pellets were resuspended in PBS and stored at -80°C.

### Transmission Electron Microscopy

Transmission electron microscope (TEM) was used to investigate the morphology of the EVs isolated by ultracentrifugation. Briefly, EVs at an optimal concentration were first placed on 400 mesh carbon/formvar-coated grids and allowed to be absorbed on formvar for a minimum of 10 min. Next, the grids (membrane side down) were transferred to a 50-μl drop of 2.5% glutaraldehyde for 5 min, after which they were transferred to a 100-μl drop of distilled water and were left to stand for 2 min. This process was repeated nine times for a total of 10 water washes. Then, the sample was loaded on the grid and stained by 4% uranyl acetate for 10 min and 1% methylcellulose for 5 min. The remaining water was removed using filter paper. Finally, the samples were viewed using a Tecnai Bio Twin TEM (FEI, Hillsboro, OR, USA), and images were obtained using an AMT CCD camera (Advanced Microscopy Techniques, Woburn, MA, USA).

### Nanoparticle Tracking Analysis

The concentration and the size distribution of EVs were analyzed by nanoparticle tracking analysis (NTA) using a ZetaView instrument (Particle Metrix, Inning am Ammersee, Germany) and the NanoSight LM10 microscope (NanoSight Ltd., Amesbury, UK) configured with a 405-nm laser. Videos were collected and analyzed using the NTA software (version 2.3) with the default setting of the minimal expected particle size, minimum track length, and blur. Each EV sample was vortexed and diluted with particle-free PBS to obtain the recommended 25–100 particles/frame of the NTA system. Five videos of typically 60-s duration were recorded to generate replicate histograms that were averaged.

### Western Blot Analysis

Cells and EVs were lysed in radioimmunoprecipitation assay (RIPA) Lysis Buffer (Beyotime Biotechnology, Shanghai, China) for 30 min on ice, and the protein concentration was measured by the Enhanced BCA Protein Assay Kit (Beyotime Biotechnology, Shanghai, China). And then, the lysates were mixed with loading buffer and heated to 100°C for 10 min. Subsequently, the samples were electrophoretically separated on an 8% sodium dodecyl sulfate–polyacrylamide gel electrophoresis(SDS-PAGE) and electro-transferred onto polyvinylidene difluoride (PVDF) membranes (Millipore, Carlsbad, CA). After blocking for 2 h at 25°C in Tris-buffered saline with 0.05% Tween-20 (TBST) and 5% non-fat dry milk, the membranes were incubated overnight at 4°C with the primary antibodies in TBST containing 5% BSA. The following antibodies were used for Western blot (WB) analysis, including anti-Alix antibody (1:1,000; ab88388; Abcam, Cambridge, MA, USA), anti-Calnexin antibody (1:200; ab238078; Abcam, Cambridge, MA, USA), anti-CD63 antibody (1:300; ab8219; Abcam, Cambridge, MA, USA), anti-EpCAM antibody (1:200; ab218448; Abcam, Cambridge, MA, USA), anti-CD9 antibody (1:300; sc-13118; Santa-Cruz Biotechnology, Santa Cruz, CA, USA), anti-beta Actin antibody (1:5,000; ab6276; Abcam, Cambridge, MA, USA), and anti-Apo antibody (1:500; ab66379; Abcam, Cambridge, MA, USA). Thereafter, the membrane was washed and immersed into horseradish peroxidase (HRP)-conjugated secondary antibodies (Jackson ImmunoResearch, Suffolk, UK) for 2 h at 25°C. Chemiluminescent detection of bands was performed using Clarity Western ECL Substrate Kit (Bio-Rad Laboratories, Inc., Hercules, CA, USA), and the signals were visualized using the Quantity One Imaging Software from Bio-Rad according to the manufacturer’s instructions. In order to quantify the levels of EpCAM-CD9-positive EVs from WB analysis and investigate the association with the chemiluminescent signals by our immunoassay, we rationally defined the EpCAM-CD9 protein density (Density_EpCAM-CD9_): Density_EpCAM-CD9_ = Density_EpCAM_ × Density_CD9_, where the value of Density_EpCAM_ and Density_CD9_ can be quantitatively obtained from WB images using Quantity One Imaging Software. This definition was based on the hypothesis that all the EVs expressing EpCAM and CD9 were sufficiently captured and detected by the antibody sets of our immunoassay, and the chemiluminescent signals of each EV captured by EpCAM antibody can be multiplied by CD9 antibody. Our results showed that Density_EpCAM-CD9_ was correlated highly with chemiluminescent signals (r = 0.8395, 95% CI: 0.6317–0.9348, P < 0.0001) (**Supplementary Figure S2A**).

### Flow Cytometry Analysis

The expression of CD9 and EpCAM on EVs were analyzed by flow cytometry as previously described (21). Briefly, EVs attached to 4 μm aldehyde/sulfate latex beads (Invitrogen, Carlsbad, CA, USA) were incubated with anti-CD9 antibodies (SAB4700092; Sigma-Aldrich, St. Louis, MO), anti-CD63 antibodies (ab1318; Abcam, Cambridge, MA, USA), anti-CD81 antibodies (ab79559; Abcam, Cambridge, MA, USA), or anti-EpCAM antibodies (ab187372; Abcam, Cambridge, MA, USA) for 30 min with rotation at 4°C followed by Alexa-488-tagged secondary antibodies (Life Technologies, Carlsbad, CA, USA) for 30 min with rotation at 4°C. Samples were detected using CytoFLEX Flow Cytometer (Beckman Coulter, Brea, CA, USA) and data were analyzed using CytExpert (Beckman Coulter, Brea, CA, USA).

### Bicinchoninic Acid Assay

According to the manufacturer’s instructions, the concentration and the protein amount of EVs were measured by bicinchoninic acid (BCA) assay using Enhanced BCA Protein Assay Kit (Beyotime Biotechnology, Shanghai, China) and a spectrophotometer (Bio-Rad Laboratories, Inc., Hercules, CA, USA) set to 562 nm.

### Urinary Creatinine and Serum Prostate-Specific Antigen

The urinary creatinine was measured with Roche-developed assays for creatinine (CRE2U, ACN 8152) using a Roche Cobas 8000 Modular Analyzer (Roche, Woerden, Netherlands) according to the manufacturer’s instructions. The automated chemiluminescent microparticle immunoassay analyzer ARCHITECT i2000 (Abbott Laboratories, Abbott Park, IL, USA) was used following the manufacturer’s protocols to determine the concentrations of PSA protein in serum samples.

### Serum Starvation and Hypoxia for Cells

Cells were seeded and cultured in RPMI 1640 medium, which contains glucose and amino acids for 24 h. The medium was discarded, and then cells were washed once with PBS to remove trace serum. The cells were further cultured in serum-free RPMI 1640 medium under normoxia (21% O_2_) to suffer serum starvation or cultured in RPMI 1640 medium supplemented with 10% FBS under hypoxic conditions (1% O_2_) to suffer hypoxia for the indicated time periods.

### Chemiluminescent Immunoassay for Extracellular Vesicle Detection

EVs were detected by a newly developed paramagnetic particle-based sandwich CLIA (**Figure 1A**).

For EVs from the cell line supernatant, 100 μl EVs were mixed with 50 μl ACE-labeled anti-CD9 antibodies (1.320 μg/ml) and biotin-labeled anti-EpCAM antibodies (4.000 μg/ml). After incubation for 1 h at 25°C, the mixtures are incubated with 50 μl turbid liquid containing 4 mg/ml avidin-coated magnetic beads for another 30 min, followed by thorough washing of the magnetic beads under an external magnetic field. Finally, magnetic beads with ACE-labeled anti-CD9 antibodies are mixed with trigger solution for chemiluminescent signal excitation. All the measurements are performed in triplicate. EV_EpCAM-CD9_ secretion index was calculated to describe the average amount of EV_EpCAM-CD9_ secreted per PC3 cell. EV_EpCAM-CD9_ secretion index = V_s_ × Con _EV_/N _cell_, where V_s_ (μl) is the volume of the PC3 cell line supernatant, Con _EV_ (particles/μl) is the concentration of EV_EpCAM-CD9_ derived by PC3 cells in the supernatant, and N_cell_ corresponds to the number of the PC3 cells.

For EVs from the urine samples, each step was the same as the EVs from the cell line supernatant, except the concentration of the ACE-labeled anti-CD9 antibodies (0.132 μg/ml). To avoid urine sampling variance, uEV_EpCAM-CD9_ concentrations were normalized by urinary creatinine. We herein rationally defined “n.u.”: n.u. = Con EV/Cr, where Con EV (g/L) corresponds to the concentration of EV_EpCAM-CD9_ in the urine samples and Cr (g/L) corresponds to the urinary creatinine of the urine samples, to compare uEV_EpCAM-CD9_ concentrations between patients with PCa and without PCa better.

### Statistical Analysis

Continuous variables were presented as mean ± standard deviation (SD) or median [interquartile range (IQR)] and compared with each other by Student’s t-test or Mann–Whitney U test. Categorical variables are presented as rate and compared using the chi-square test or the Fisher’s exact test. Receiver operating characteristic (ROC) curve was used to evaluate the diagnostic performance of EpCAM-CD9-positive EVs, PSA, and models. Decision curve analysis (DCA) was used to compare the diagnostic benefits of different biomarkers and models for PCa. P-values lower than 0.05 were considered statistically significant. All analyses were undertaken with GraphPad Prism version 8.0, SPSS Statistics 20, and R version 2.10.1 (R Foundation for Statistical Computing; http://www.R-project.org).

## Results

### Characterization of Extracellular Vesicles From the Prostate Cell Line PC3

In this study, we used PC3-derived EVs to construct and optimize the CLIA. Standard characterization of EVs was performed using TEM, NTA, and WB analysis (**Figures 1B–D**). EVs showed characteristic cup-shaped morphology under TEM and showed a mean size of 175.9 ± 6.3 nm (standard error; SD: 78.6 ± 11.1 nm) by NTA. The EV fraction from PC3 was enriched in CD63, CD9, and ALIX, the common biomarkers of EVs, but did not contain calnexin and APO, the negative control of EVs, compared to the PC3 cell lysates (20). In addition, PC3-derived EVs were positive for EpCAM, an epithelial cell marker. Moreover, flow cytometry assay identified that approximately 80% of EVs released by PC3 carried EpCAM and CD9 (**Figures 1E–G**). These results indicated that EpCAM and CD9 were enriched on the membrane of EVs from the prostate cell line PC3 and EVs can be effectively captured by anti-EpCAM antibody-conjugated magnetic beads and successfully detected by ACE-labeled anti-CD9 antibodies.

### Ultrasensitive Detection of EpCAM-CD9-Positive Extracellular Vesicles by Chemiluminescent Immunoassay

**Figure 2:**
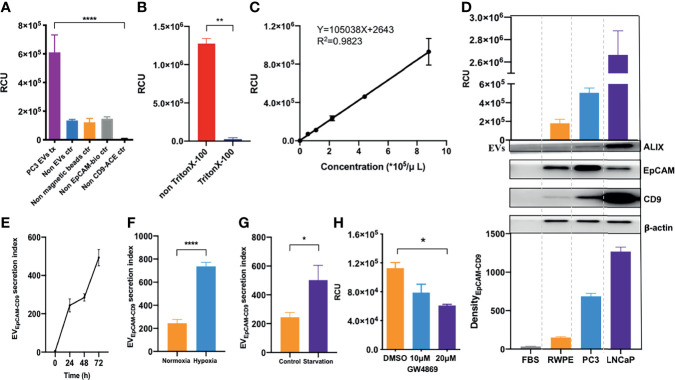
EV_EpCAM-CD9_ is ultrasensitively detected by chemiluminescent immunoassay and oversecreted under simulated tumor microenvironment. **(A)** Groups of PC3 EVs, non-EVs, non-streptavidin-labeled magnetic beads, non-biotin-labeled anti-EpCAM antibodies, and non-ACE-labeled anti-CD9 antibodies were detected by our assay. **(B)** EVs were penetrated by Triton X-100. **(C)** A standard curve was for EVs from cell line supernatant quantification using our EV assay. **(D)** EVs derived from FBS, BPH cell line RWPE-1, and human prostate cancer cell lines PC3 and LNCaP were detected by our EV assay and WB. **(E)** The changes of EV_EpCAM-CD9_ secretion index during the growth of PC3 cells. **(F)** The changes of EV_EpCAM-CD9_ secretion index when the PC3 cells were cultured under hypoxia. **(G)** The changes of EV_EpCAM-CD9_ secretion index when the PC3 cells were cultured under serum starvation. **(H)** The changes of EV_EpCAM-CD9_ secreted by PC3 cells with the treatment of 10 and 20 μM GW4869. RCU, relative chemiluminescent unit; EVs, extracellular vesicles; FBS, fetal bovine serum; EVEpCAM-CD9, EpCAM-CD9-positive extracellular vesicles. *P < 0.05,**P < 0.01,****P < 0.0001.

We performed an ultrasensitive CLIA to quantify EV_EpCAM-CD9_ (**Figure 1A**). Noteworthy, although several conventional surface markers (e.g., CD9, CD63, and CD81) are used for EV analysis, we selected CD9 as our detection antibody for EVs. The expression of CD9, CD63, CD81, and EpCAM on PC3-derived EVs were analyzed by flow cytometry in our study, showing that EVs carrying CD9, CD63, CD81, and EpCAM accounted for 81.42%, 82.08%, 67.19%, and 79.59% of total PC3-derived EVs, respectively. Practically, the CLIA employing ACE-labeled anti-CD9 antibody exhibited a superior performance over ACE-labeled CD63 antibody or ACE-labeled CD81 antibody (data not shown). This assay exhibited remarkable chemiluminescent signals for PC3-derived EVs, while the four control groups (non-EVs, non-streptavidin-labeled magnetic beads, non-biotin-labeled anti-EpCAM antibodies, and non-ACE-labeled anti-CD9 antibodies) presented negligible chemiluminescent signals (**Figure 2A**). By contrast, a significant reduction in the relative chemiluminescent unit (RCU) was observed after the addition of Triton X-100, a detergent to lyse EVs (**Figure 2B**) (22). These results strongly demonstrated the feasibility of the assay for selectively detecting EVs.

Next, we systematically optimized the reaction conditions of the EV assay, including the concentration of streptavidin-labeled magnetic beads, biotin-labeled anti-EpCAM antibodies, ACE-labeled anti-CD9 antibodies, and reaction time (**Supplementary Figures S1A–D**). To further investigate the quantitative performance of the EV assay, isolated PC3-derived EVs by ultracentrifugation were quantified using the EV assay based on the concentrations obtained by NTA. As shown in **Figure 2C**, the RCU value was found to greatly depend on the concentration of EVs, with a good linearity range ranging from 5.50 × 10^4^ to 8.80 × 10^5^ particles/μl (R^2^ = 0.9823). The limit of detection (LOD) calculated as three times of SD above the background (negative control) was 2.86 × 10^4^ particles/μl. Moreover, PC3-derived EVs were quantified by a standard procedure of a recovery test to evaluate the accuracy of the EV assay. The recovery rates of low, medium, and high concentrations of EVs were 85.45%, 95.45%, and 101.65%, respectively (**Supplementary Table S2**, left panels). In addition, three different concentrations of EVs were tested to evaluate the repeatability of the EV assay. The intra-assay coefficient of variation (intra-CV) and the inter-assay coefficient of variation (inter-CV) were less than 10% and 20%, respectively (**Supplementary Table S2**, left panels). The above results suggested an excellent analytical performance of our EV assay.

Then, we asked whether the EV_EpCAM-CD9_ could be used to infer the prostatic cell types, e.g., PCa cell lines (PC3 and LNCaP) and benign prostate epithelial cell line (RWPE-1). Hence, we obtained the EVs from the culture supernatant by ultracentrifugation and quantified the concentrations by our assay. As shown in **Figure 2D**, the concentrations of EVs derived from human PCa cell lines such as PC3 and LNCaP were significantly higher than that of the BPH cell line RWPE-1, which were consistent with the EV Density_EpCAM-CD9_ from corresponding cell lines. Moreover, FBS-derived EVs exhibited negligible chemiluminescent signals in the assay. These results revealed that the concentration of EV_EpCAM-CD9_ can be a potential indicator for distinguishing cancerous cells from normal ones.

### EV_EpCAM-CD9_ Are Oversecreted by Prostate Cancer Cells Under Simulated Tumor Microenvironment

In the course of tumor expansion, cancer cells within the tumor microenvironment often have restricted access to nutrients and oxygen and thus were subjected to starvation and hypoxia (23). Previous reports have demonstrated that the levels of EVs carrying tumor-related proteins can be significantly elevated under such microenvironment, contributing to the regulation of tumor microenvironment, thus promoting tumor initiation, progression, and metastasis (24). However, EV_EpCAM-CD9_ derived from PCa cells under tumor microenvironment, which may be diagnostically beneficial in reflecting the pathological stage during PCa development, remained unknown.

Herein, we defined EV_EpCAM-CD9_ secretion index to describe the average amount of EV_EpCAM-CD9_ secreted per PC3 cell. As shown in **Figure 2E**, the EV_EpCAM-CD9_ secretion index was gradually elevated at the early stage of cell growth due to the initial activation of the cells in the latent phase. At 24–48 h, the PC3 cells entered the logarithmic growth phase, and the equative rate of increase between the amount of EV_EpCAM-CD9_ and PC3 cells resulted in a constant EV_EpCAM-CD9_ secretion index. Interestingly, however, when the cell reached the stationary phase after 48 h, the EV_EpCAM-CD9_ secretion index started increasing again. This may be a result of the inadequate living conditions in the microenvironment. Accordingly, we investigated the impact of some conditions (e.g., hypoxia and serum starvation) involved in such microenvironment on the EV_EpCAM-CD9_ secretion index. We observed higher EV_EpCAM-CD9_ secretion indexes when the PC3 cells were cultured under hypoxia and serum starvation compared with the controls (**Figures 2F, G** **)**. Additionally, this trend can be reversed upon the treatment of the EV biogenesis inhibitor, such as GW4869 (**Figure 2H**). These results strongly support our hypothesis that EV_EpCAM-CD9_ can be a potential indicator in revealing the pathological status of PCa.

### Urinary EpCAM-CD9-Positive Extracellular Vesicle Is a Biomarker for Prostate Cancer Diagnosis

**Figure 3:**
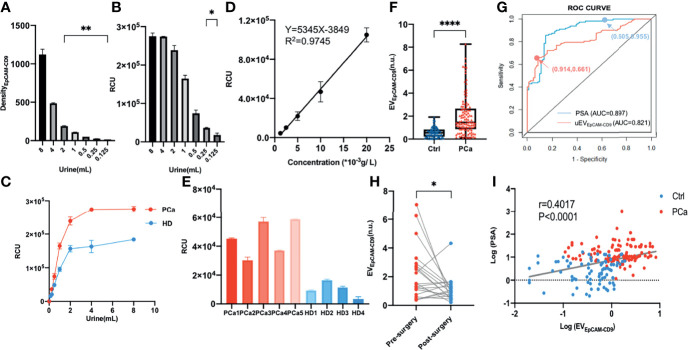
Urinary EpCAM-CD9-positive EV is a potential biomarker for PCa diagnosis. **(A)** The uEV_EpCAM-CD9_ in different urine volumes was detected by WB. **(B)** The uEV_EpCAM-CD9_ from different urine volumes was detected by our chemiluminescent immunoassay. **(C)** The uEV_EpCAM-CD9_ of the pooled samples from PCa and healthy controls were detected by EV assay. **(D)** A standard curve was for urinary EV quantification using our EV assay. **(E)** The uEV_EpCAM-CD9_ from nine randomly selected donors including five PCa and four HDs was assayed by the chemiluminescent immunoassay. **(F)** The levels of uEV_EpCAM-CD9_ was observed from men with PCa (n = 112) and without PCa (n = 81). **(G)** The ROC curve of uEV_EpCAM-CD9_ and PSA. **(H)** The uEV_EpCAM-CD9_ was detected before and after prostatectomy in 20 PCa patients. **(I)** The correlation between the uEV_EpCAM-CD9_ and PSA. Density_EpCAM-CD9_, EpCAM-CD9 protein density; RCU, relative chemiluminescent unit; EV_EpCAM-CD9_, EpCAM-CD9-positive extracellular vesicles; Ctrl, control; PCa, prostate cancer; ROC, receiver operating characteristic; uEVEpCAM-CD9, urinary EpCAM-CD9-positive extracellular vesicles; PSA, prostate-specific antigen; AUC, area under the curve; HD, healthy donor. *P < 0.05,**P < 0.01,****P < 0.0001.

Urine can harbor PCa cell-derived EVs, as mentioned above. We thus investigated whether the urinary EpCAM-CD9-positive EVs (uEV_EpCAM-CD9_) can be detected using our EV assay. As shown in **Supplementary Figure S2B**, the protein profile from urine revealed the presence of EpCAM and CD9-positive EVs in PCa. Using a well-adopted EV protein assay, WB, uEV_EpCAM-CD9_ from less than 2 ml of urine volume was almost undetectable (**Figure 3A**). However, the uEV_EpCAM-CD9_ from even down to 125 μl of urine volume could be successfully detected by our CLIA, and the levels of uEV_EpCAM-CD9_ in the same urine volume were statistically distinguishable between the pooled samples from PCa and healthy controls (**Figures 3B, C** **)**. In view of the significant differences between the cell supernatant and urine in the concentration and proportion of EV_EpCAM-CD9_, we optimized the methodology again. Additionally, in the clinical laboratory, it is not suitable to quantify EVs by NTA due to the requirement of the specialized equipment. And NTA may be biased toward certain particle size ranges (especially 50–150 nm), and large EVs (>400 nm) and very small EVs (<50 nm) are not well quantified by NTA. We thus used a simple and low-cost protein assay, BCA, as an alternative for EV quantification (25). As described in the *Materials and Methods*, we optimized the concentration of CD9 antibody and restructured the standard curve and corresponding performance evaluation. As shown in **Figure 3D**, the RCU value was found to greatly depend on the concentration of EVs, with a good linearity range ranging from 1.25 × 10^-3^ to 20.00 × 10^-3^ g/L (R^2^ = 0.9745) and a low detection limit, 0.60 × 10^-3^ g/L. The recovery test and repeatability test both performed excellently especially at the low level of uEV_EpCAM-CD9_ (**Supplementary Table S2**, right panels). Furthermore, uEV_EpCAM-CD9_ from nine randomly selected donors including five PCa and four HDs was assayed by the CLIA and WB, which suggested a significant elevation of uEV_EpCAM-CD9_ in PCa compared with HD (**Figure 3E** and **Supplementary Figures S2C, D**).

In the validation experiment, urine samples from a total of 193 participants were further enrolled, including 112 PCa patients, 55 BPH patients, and 26 HDs. Complete datasets were available in 193 men who underwent the first transrectal ultrasound (TRUS)-guided prostate biopsy, and the histologic subtypes of all the 112 PCa patients were identified as prostate adenocarcinoma and without any metastatic sites confirmed by computed tomography examinations. The clinical characteristics of all the participants were listed in **Supplementary Table S1**. A remarkably higher level of uEV_EpCAM-CD9_ was observed from men with PCa (1.46, IQR 0.86-2.66) than men without PCa (0.55, IQR 0.22-0.84) (**Figure 3F**). ROC curve showed that the diagnostic sensitivity and specificity of uEV_EpCAM-CD9_ was 66.07% and 91.36%, respectively (cutoff value: 1.130), and the area under the curve (AUC) was 0.821 (P < 0.0001), while the diagnostic sensitivity and specificity of PSA was 95.54% and 60.49%, respectively (cutoff value: 4.015), and the AUC was 0.897 (P < 0.0001) (**Figure 3G**). Moreover, there was a statistically significant correlation between uEV_EpCAM-CD9_ and Gleason grades in PCa patients (r = 0.215, 95% CI: 0.025–0.389, P = 0.023). Significant decreases of uEV_EpCAM-CD9_ were observed after prostatectomy in 20 PCa patients (**Figure 3H**). It also showed that uEV_EpCAM-CD9_ levels were positively associated with PSA (r = 0.402, 95% CI: 0.272–0.517, P < 0.0001), which was an important indicator for the diagnosis of PCa (**Figure 3I**).

### A Multivariate Diagnostic Model Based on uEV_EpCAM-CD9_ for Prostate Cancer

Due to the results that uEV_EpCAM-CD9_ has high specificity and low sensitivity, while PSA is just the opposite (**Figure 3G**), we consider building a model combining uEV_EpCAM-CD9_ and PSA to better diagnose PCa. The training dataset (n = 116) and validation dataset (n = 77) had an even distribution in patient characteristics (**Table 1**). The predictive value of the uEV_EpCAM-CD9_ was analyzed using a logistic regression model. The odds ratio (OR) for each clinical factor and/or covariate in training sets was assessed by univariate logistic regression modeling. Age, uEV_EpCAM-CD9_, PSA, fPSA, f/T PSA, prostate volume (PV), and prostate-specific antigen density (PSAD) were statistically significant predictors of PCa (P < 0.001) on univariate logistic regression analysis (**Table 2**, left panels). Then, we compared varieties of multivariate diagnostic models employing different combinations of the variables assessed by their AUC in ROC curve analysis variables (**Supplementary Table S3**). The optimal multivariate model for diagnosing PCa should be selected on the basis of the complexity (numbers of variables) and prediction efficiency (AUC); we rationally selected the multivariate model containing the variable age, smoking, drinking, family history, BMI, uEV_EpCAM-CD9_, PSA, and PV as the final diagnostic model. The OR of each variable from the multivariate logistic regression analysis was presented in **Table 2** (right panels).

**Table 1:** Baseline characteristics of the training and validation cohorts.

Variable	Training Set (n = 116)			Validation Set (n = 77)		P value
	Men with PCa (n = 69)	Men without PCa (n = 47)	P value	Men with PCa (n = 43)	Men without PCa (n = 34)	
	Median (IQR) or n (%)	Median (IQR) or n (%)		Median (IQR) or n (%)	Median (IQR) or n (%)	
Age (years)	72 (66–76)	64 (51–70)	<0.0001	71 (64–74)	64 (55–73)	0.018
Smoking			0.014			0.127
Yes	30 (43.5)	10 (21.3)		20 (46.5)	10 (29.4)	
No	39 (56.5)	37 (78.7)		23 (53.5)	24 (70.6)	
Drinking			0.026			0.229
Yes	30 (43.5)	11 (23.4)		17 (39.5)	9 (26.5)	
No	39 (56.5)	36 (76.6)		26 (60.5)	25 (73.5)	
Family history			0.167			0.428
Yes	8 (11.6)	2 (4.3)		3 (7.0)	1 (2.9)	
No	61 (88.4)	45 (95.7)		40 (93.0)	33 (97.1)	
BMI (kg/m²)	23.88 (22.00–26.03)	22.23 (21.29–24.62)	0.012	23.30 (21.80–25.08)	22.78 (21.72–24.14)	0.538
Gleason score			<0.0001			<0.0001
6	12 (17.4)	NA		5 (11.6)	NA	
7	32 (46.4)	NA		17 (39.5)	NA	
8	12 (17.4)	NA		9 (20.9)	NA	
9–10	13 (18.8)	NA		12 (27.9)	NA	
CEA (ng/ml)	2.6 (2.1–3.5)	2.1 (1.4–3.0)	0.019	2.2 (1.8–3.2)	2.0 (1.5–3.0)	0.228
AFP (ng/ml)	2.6 (1.8–3.5)	2.5 (1.8–3.6)	0.833	2.8 (1.7–3.2)	2.5 (1.6–3.0)	0.285
CA125 (U/ml)	9.9 (7.1–12.8)	11.5 (7.1–13.9)	0.389	11.3 (9.1–14.5)	11.2 (5.5–12.8)	0.327
CA199 (U/ml)	7.4 (4.1–11.3)	7.1 (4.0–11.6)	0.884	6.2 (4.7–11.9)	5.9 (3.7–11.1)	0.432
EpCAM-CD9-positive EV concentration (n.u)	1.38 (0.56–2.48)	0.53 (0.31–0.84)	<0.0001	1.57 (1.15–3.08)	0.58 (0.16–0.91)	<0.0001
PSA (ng/ml)	10.9820 (7.3635–22.0090)	2.6780 (0.8072–5.5960)	<0.0001	14.8340 (9.5180–29.9020)	2.7025 (1.0583–8.3488)	<0.0001
fPSA (ng/ml)	1.5390 (1.0730–3.0710)	0.6777 (0.3045–1.1920)	<0.0001	2.1080 (0.8620–3.2330)	0.7826 (0.2259–1.6068)	<0.0001
f/T PSA	0.13 (0.09–0.20)	0.22 (0.18–0.34)	<0.0001	0.11 (0.08–0.19)	0.23 (0.19–0.29)	<0.0001
PV (cm³)	63.00 (44.93–109.35)	66.58 (24.00–107.04)	0.556	54.71 (47.23–77.76)	50.34 (24.00–110.83)	0.785
PSAD (ng/ml²)	0.17 (0.08–0.45)	0.04 (0.02–0.06)	<0.0001	0.31 (0.17–0.43)	0.05 (0.03–0.07)	<0.0001

BMI, body mass index; EV, extracellular vesicle; PSA, prostate-specific antigen; fPSA, free prostate-specific antigen; f/T PSA, free/total prostate-specific antigen; PV, prostate volume; PSAD, prostate-specific antigen density; PCa, prostate cancer; IQR, interquartile range; NA, not applicable.

**Table 2:** Univariate analysis and multivariate analysis of potential predictors of PCa.

Variable	Univariate analysis		Multivariate analysis	
	OR (95% CI)	P value	OR (95% CI)	P value
Age (years)	1.090 (1.053–1.129)	<0.001	1.019 (0.962–1.080)	0.515
Smoking	2.460 (1.313–4.607)	0.005	0.579 (0.148–2.268)	0.433
Drinking	2.205 (1.176–4.138)	0.014	1.690 (0.462–6.178)	0.428
Family history	2.832 (0.764–10.498)	0.119	4.386 (0.452–42.528)	0.202
BMI (≥24 kg/m² *vs*. <24 kg/m²)	2.186 (1.188–4.019)	0.012	1.312 (0.433–3.976)	0.631
CEA (ng/ml)	1.227 (0.988–1.523)	0.064		
AFP (ng/ml)	1.109 (0.937–1.313)	0.228		
CA125 (U/ml)	1.016 (0.965–1.069)	0.545		
CA199 (U/ml)	1.002 (0.980–1.025)	0.836		
Log EpCAM-CD9-positive EV concentration (n.u)	15.392 (6.377–37.149)	<0.001	28.745 (6.438–128.346)	<0.001
PSA (ng/ml)				
<4	Reference	<0.001	Reference	<0.001
4–10	15.200 (5.301–43.581)	<0.001	33.292 (6.105–181.543)	<0.001
>10	73.600 (23.220–233.284)	<0.001	169.450 (25.652–1119.355)	<0.001
fPSA (ng/ml)	2.007 (1.470–2.742)	<0.001		
f/T PSA	0.000 (0.000–0.003)	<0.001		
PV (cm³)				
<36	Reference	<0.001	Reference	0.001
36–48	6.462 (2.079–20.086)	0.001	1.384 (0.173–11.083)	0.760
48–72	9.333 (2.079–24.838)	<0.001	3.352 (0.489–22.973)	0.218
72–108	2.741 (0.981–7.661)	0.054	0.203 (0.025–1.636)	0.134
>108	2.234 (0.961–5.194)	0.062	0.088 (0.012–0.633)	0.016
PSAD (≥0.15 ng/ml² *vs*. <0.15 ng/ml²)	68.402 (15.964–293.082)	<0.001		

PCa, prostate cancer; BMI, body mass index; EV, extracellular vesicle; PSA, prostate-specific antigen; fPSA, free prostate-specific antigen; f/T PSA, free/total prostate-specific antigen; PV, prostate volume; PSAD, prostate-specific antigen density; OR, odds ratio; CI, confidence interval.

**Figure 4:**
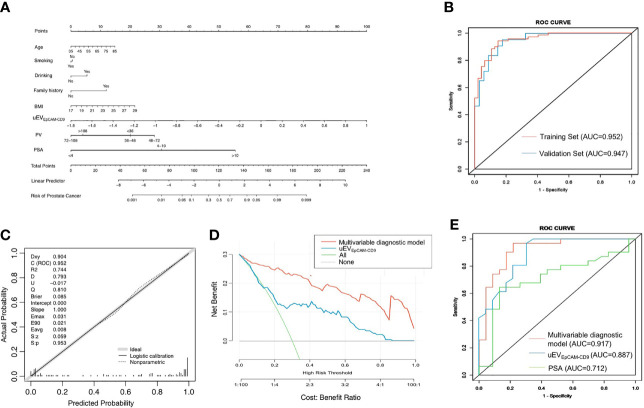
A multivariate diagnostic model based on uEV_EpCAM-CD9_ for PCa. **(A)** The nomogram was constructed according to the results of multivariate logistic regression. **(B)** The ROC curve analysis of the multivariable diagnostic model in the training set and validation set. **(C)** The multivariable diagnostic model was calibrated in the internal validations. **(D)** The decision curve analysis of the multivariable diagnostic model and uEV_EpCAM-CD9_. **(E)** The diagnostic performance of the model, uEV_EpCAM-CD9_, and PSA in patients with PSA gray zone (4–10 ng/ml) including 23 PCa and 31 BPH. BMI, body mass index; uEV_EpCAM-CD9_, Log urinary EpCAM-CD9-positive extracellular vesicles concentration (n.u); PV, prostate volume; PSA, prostate-specific antigen; ROC, receiver operating characteristic; AUC, area under the curve.

The nomogram was constructed according to the results of multivariate logistic regression (**Figure 4A**). In the ROC curve analysis, the AUC of the combined PCa diagnostic model was increased to 0.952 in the training set (**Figure 4B**). Moreover, the multivariate diagnostic model was perfectly in the internal validations, as the calibration curve showed good agreement between prediction and observation (**Figure 4C**). On DCA, by combining uEV_EpCAM-CD9_ with other clinical parameters, the combination model to predict PCa added more clinical overall benefit than that of uEV_EpCAM-CD9_ only (**Figure 4D**). When applied to the validation test, the model achieved an AUC of 0.947 (P < 0.0001) (**Figure 4B**). The AUC value revealed the high performance of PCa diagnosis using the combined nomogram. Additionally, in patients with PSA gray zone (4–10 ng/ml) including 23 PCa and 31 BPH, the model based on uEV_EpCAM-CD9_ showed a better diagnostic performance (AUC = 0.917, P < 0.0001) than the uEV_EpCAM-CD9_ only (AUC = 0.887, P < 0.0001) and the traditional biomarkers PSA (AUC = 0.712, P = 0.0018) (**Figure 4E**).

## Discussion

EVs represent a rich source of information in many liquid biopsy samples, including plasma, serum, and urine, since they are abundantly released by most tumors and are relatively stable in the biological fluids, whereas cell-free nucleic acids suffer rapid degradation and are always presented at low concentration (26). PCa cell-derived EVs in urine have been extensively studied recently and regarded as novel biomarkers for cancer diagnosis. However, the major concern about the use of EVs as biomarkers in the clinical laboratory is the difficulties in the characterization of EVs. Consequently, there will be essential interest in developing standardized sampling and analytical techniques for reliable and reproducible measurements. CLIA is a non-isotopic immunological technique that is increasingly used in ultramicroanalysis of biological substances owing to extreme sensitivity, high specificity, good reproducibility, and simplicity (27, 28). In this study, we proposed a chemiluminescent quantitative immunoassay of uEV_EpCAM-CD9_, requiring only a small volume of urine (125 μl) to perform an EV analysis, which is superior to WB and flow cytometry (29). The extremely low LOD of EpCAM revealed that it was possible to detect other non-abundant proteins on EVs by employing multiple antibody sets. Furthermore, the use of CLIA embodies the superiority that could be fully automated to reduce operator errors and bias and enhance its potential for clinical translation.

EpCAM (also known as CD326) is deemed as a cancer-associated marker, as it is always overexpressed in many human adenocarcinomas and squamous cell carcinomas (30). Besides, this expression often closely correlates with the epithelial–mesenchymal transition (EMT)-regulating tumor invasion and metastasis (31, 32): the tumor cells have been observed to undergo loss of EpCAM expression during EMT and release a large number of EpCAM-enriched EVs simultaneously (32, 33). The source and the underlying functions of these EVs in PCa, however, remain unknown. It has been suggested that the cancerous cells will proliferate more rapidly due to the dysregulated cell cycle resulting in a state of oxygen and nutrient deprivation, and adaptation to such microenvironments is pivotal to tumor growth (34). There is good evidence that many signaling pathways are involved to help the cells escape from stresses such as hypoxia and nutrient deprivation and determine cell growth, promotion, metastasis, hormone-refractory progression, and treatment outcome (35, 36). Additionally, previous studies have reported that higher numbers of EVs were secreted by cancer cells to offer a survival advantage to cells and promote cancer progression under hypoxia and serum starvation (37, 38). These EVs usually promoted the PCa aggressiveness by adhesion junction proteins that could enhance invasiveness and induce microenvironment changes (39). Thus, such mechanisms may account for the elevated levels of PCa cell-derived EV_EpCAM-CD9_ under simulated tumor microenvironment (such as hypoxia and serum starvation), as well as in PCa patients.

However, uEV_EpCAM-CD9_ was not prostate-specific; that is, it may be over-released by other urogenital tumors such as bladder and kidney and other non-urological cancers. Recalling that the levels are commonly very low in HDs and patients with BPH, our multivariate model employing uEV_EpCAM-CD9_, prostate tissue-specific protein (PSA), and other clinical parameters showed an enhanced diagnostic performance both in sensitivity and specificity. We also envision that by combining other cancer biomarkers, such as metabolites, RNAs or genetic signatures and medical imaging data could further provide more precise information regarding PCa diagnosis and localization.

Another limitation is the number of samples studied (n = 193). We only evaluated the diagnostic value of uEV_EpCAM-CD9_ in PCa, and it has not been evaluated in depth in other aspects, e.g., as a predictor in the development of castration-resistant prostate cancer (CRPC), an indicator for successful radiotherapy and chemotherapy. Besides, while EV_EpCAM-CD9_ can be released from different types of epithelial cancers and the diagnostic performance of uEV_EpCAM-CD9_ in these cancers remains poorly investigated, further large-scale studies will be warranted to fully evaluate the potential applications of uEV_EpCAM-CD9_ with regard to the diagnosis of varieties of cancers.

## Conclusions

Urinary EpCAM-CD9-positive EVs were successfully quantified by our laboratory-developed CLIA, requiring only a small volume of urine (125 μl) to perform an EV analysis. Using this assay, we achieve a notable diagnostic performance by constructing a multivariate diagnostic model based on uEV_EpCAM-CD9_ and a tissue-specific biomarker PSA. Further validation studies are warranted and should also investigate before its clinical value can be confidently affirmed. In the future, this model may potentially be used to better select patients for prostate TRUS biopsy.

## Data Availability Statement

The raw data supporting the conclusions of this article will be made available by the authors without undue reservation.

## Ethics Statement

The studies involving human participants were reviewed and approved by the Second Affiliated Hospital of Zhejiang University School of Medicine Ethical Committee. The patients/participants provided their written informed consent to participate in this study.

## Author Contributions

YD, YW, XW, and ZT planned the project, performed the research, analyzed the data, and wrote the article. YC, YS, and DW collected clinical samples. PY and LZ performed statistical analysis. ZL, YP, and GZ collected patient information and analyzed the data. All authors contributed to the article and approved the submitted version.

## Funding

This study was supported by grants from the National Natural Science Foundation of China Youth Science Foundation Project (Grant no. 81902156) and Zhejiang Provincial Natural Science Foundation of China (Grant no. LQ21H160016).

## Conflict of Interest

The authors declare that the research was conducted in the absence of any commercial or financial relationships that could be construed as a potential conflict of interest.

## Publisher’s Note

All claims expressed in this article are solely those of the authors and do not necessarily represent those of their affiliated organizations, or those of the publisher, the editors and the reviewers. Any product that may be evaluated in this article, or claim that may be made by its manufacturer, is not guaranteed or endorsed by the publisher.

## Abbreviations

EpCAM, epithelial cell adhesion molecule; PCa, prostate cancer; PSA, prostate-specific antigen; EVs, extracellular vesicles; uEV_EpCAM-CD9_, urinary EpCAM-CD9-positive extracellular vesicles; AUC, area under the curve; TRUS, transrectal ultrasound; *PCA3*, prostate cancer antigen-3; *TMPRSS2*, transmembrane protease serine-2; *GSTP1*, glutathione S-transferase; uEVs, urinary extracellular vesicles; CLIA, chemiluminescent immunoassay; ACE, acridinium ester; EV_EpCAM-CD9_, EpCAM-CD9-positive extracellular vesicles; FBS, fetal bovine serum; BPH, benign prostatic hyperplasia; HD, healthy donor; PBS, phosphate-buffered saline; TEM, transmission electron microscope; NTA, nanoparticle tracking analysis; RIPA, radioimmunoprecipitation assay; SDS-PAGE, sodium dodecyl sulfate-polyacrylamide gel electrophoresis; PVDF, polyvinylidene difluoride; TBST, Tris-buffered saline with Tween-20; WB, western blot; HRP, horseradish peroxidase; Density_EpCAM-CD9_, EpCAM-CD9 protein density; BCA, bicinchoninic acid; SD, standard deviation; IQR, interquartile range; ROC, receiver operating characteristic; DCA, decision curve analysis; RCU, relative chemiluminescent unit; LOD, limit of detection; intra-CV, intra-assay coefficient of variation; inter-CV, inter-assay coefficient of variation; CI, confidence interval; OR, odds ratio; fPSA, free prostate-specific antigen; f/T PSA, free/total prostate-specific antigen; PV, prostate volume; PSAD, prostate-specific antigen density; BMI, body mass index; EMT, epithelial–mesenchymal transition; CRPC, castration-resistant prostate cancer.

## References

[1] Sung H Ferlay J Siegel RL Laversanne M Soerjomataram I Jemal A. Global Cancer Statistics 2020: GLOBOCAN Estimates of Incidence and Mortality Worldwide for 36 Cancers in 185 Countries. CA Cancer J Clin (2021) 71:209–49. doi: 10.3322/caac.21660 33538338

[2] Martin R Donovan J Turner E Metcalfe C Young G Walsh E. Effect of a Low-Intensity PSA-Based Screening Intervention on Prostate Cancer Mortality: The CAP Randomized Clinical Trial. JAMA (2018) 319:883–95. doi: 10.1001/jama.2018.0154 PMC5885905 29509864

[3] Brassell SA Kao TC Sun L Moul JW. Prostate-Specific Antigen *Versus* Prostate-Specific Antigen Density as Predictor of Tumor Volume, Margin Status, Pathologic Stage, and Biochemical Recurrence of Prostate Cancer. Urology (2005) 66:1229–33. doi: 10.1016/j.urology.2005.06.106 16360448

[4] Sanda M Feng Z Howard D Tomlins S Sokoll L Chan D. Association Between Combined TMPRSS2:ERG and PCA3 RNA Urinary Testing and Detection of Aggressive Prostate Cancer. JAMA Oncol (2017) 3:1085–93. doi: 10.1001/jamaoncol.2017.0177 PMC5710334 28520829

[5] Crawford E Rove K Trabulsi E Qian J Drewnowska K Kaminetsky J. Diagnostic Performance of PCA3 to Detect Prostate Cancer in Men With Increased Prostate Specific Antigen: A Prospective Study of 1,962 Cases. J Urol (2012) 188:1726–31. doi: 10.1016/j.juro.2012.07.023 22998901

[6] Laxman B Tomlins S Mehra R Morris D Wang L Helgeson B. Noninvasive Detection of TMPRSS2:ERG Fusion Transcripts in the Urine of Men With Prostate Cancer. Neoplasia (New York NY) (2006) 8:885–8. doi: 10.1593/neo.06625 PMC1715928 17059688

[7] Van Neste L Partin A Stewart G Epstein J Harrison D Van Criekinge W. Risk Score Predicts High-Grade Prostate Cancer in DNA-Methylation Positive, Histopathologically Negative Biopsies. Prostate (2016) 76:1078–87. doi: 10.1002/pros.23191 PMC5111760 27121847

[8] Davey M Benzina S Savoie M Breault G Ghosh A Ouellette R. Affinity Captured Urinary Extracellular Vesicles Provide mRNA and miRNA Biomarkers for Improved Accuracy of Prostate Cancer Detection: A Pilot Study. Int J Mol Sci (2020) 21:8330. doi: 10.3390/ijms21218330 PMC7664192 33172003

[9] Kohaar I Chen Y Banerjee S Borbiev T Kuo H Ali A. A Urine Exosome Gene Expression Panel Distinguishes Between Indolent and Aggressive Prostate Cancers at Biopsy. J Urol (2021) 205:420–5. doi: 10.1097/ju.0000000000001374 32945736

[10] Merchant M Rood I Deegens J Klein J. Isolation and Characterization of Urinary Extracellular Vesicles: Implications for Biomarker Discovery. Nat Rev Nephrol (2017) 13:731–49. doi: 10.1038/nrneph.2017.148 PMC5941934 29081510

[11] Pisitkun T Shen R Knepper M. Identification and Proteomic Profiling of Exosomes in Human Urine. Proc Natl Acad Sci USA (2004) 101:13368–73. doi: 10.1073/pnas.0403453101 PMC516573 15326289

[12] Gonzales P Pisitkun T Hoffert J Tchapyjnikov D Star R Kleta R. Large-Scale Proteomics and Phosphoproteomics of Urinary Exosomes. J Am Soc Nephrol: JASN (2009) 20:363–79. doi: 10.1681/asn.2008040406 PMC2637050 19056867

[13] Moon P You S Lee J Hwang D Baek M. Urinary Exosomes and Proteomics. Mass Spectromet Rev (2011) 30:1185–202. doi: 10.1002/mas.20319 21544848

[14] Gires O Pan M Schinke H Canis M Baeuerle P. Expression and Function of Epithelial Cell Adhesion Molecule EpCAM: Where Are We After 40 Years? Cancer Metastasis Rev (2020) 39:969–87. doi: 10.1007/s10555-020-09898-3 PMC7497325 32507912

[15] Fagotto F Aslemarz A. EpCAM Cellular Functions in Adhesion and Migration, and Potential Impact on Invasion: A Critical Review. Biochim Biophys Acta Rev Cancer (2020) 1874:188436. doi: 10.1016/j.bbcan.2020.188436 32976980

[16] Miller M Doyle G Terstappen L. Significance of Circulating Tumor Cells Detected by the CellSearch System in Patients With Metastatic Breast Colorectal and Prostate Cancer. J Oncol (2010) 2010:617421. doi: 10.1155/2010/617421 20016752 PMC2793426

[17] Pu K Li C Zhang N Wang H Shen W Zhu Y. Epithelial Cell Adhesion Molecule Independent Capture of Non-Small Lung Carcinoma Cells With Peptide Modified Microfluidic Chip. Biosensors Bioelectron (2017) 89:927–31. doi: 10.1016/j.bios.2016.09.092 27818051

[18] Wei P Wu F Kang B Sun X Heskia F Pachot A. Plasma Extracellular Vesicles Detected by Single Molecule Array Technology as a Liquid Biopsy for Colorectal Cancer. J Extracell Vesicles (2020) 9:1809765. doi: 10.1080/20013078.2020.1809765 32944195 PMC7480466

[19] Sunkara V Kim C Park J Woo H Kim D Ha H. Fully Automated, Label-Free Isolation of Extracellular Vesicles From Whole Blood for Cancer Diagnosis and Monitoring. Theranostics (2019) 9:1851–63. doi: 10.7150/thno.32438 PMC6485293 31037143

[20] Théry C Witwer K Aikawa E Alcaraz M Anderson J Andriantsitohaina R. Minimal Information for Studies of Extracellular Vesicles 2018 (MISEV2018): A Position Statement of the International Society for Extracellular Vesicles and Update of the MISEV2014 Guidelines. J Extracell Vesicles (2018) 7:1535750. doi: 10.1080/20013078.2018.1535750 30637094 PMC6322352

[21] Wang Y Liu Z Wang X Dai Y Li X Gao S. Rapid and Quantitative Analysis of Exosomes by a Chemiluminescence Immunoassay Using Superparamagnetic Iron Oxide Particles. J BioMed Nanotechnol (2019) 15:1792–800. doi: 10.1166/jbn.2019.2809 31219017

[22] Osteikoetxea X Sódar B Németh A Szabó-Taylor K Pálóczi K Vukman K. Differential Detergent Sensitivity of Extracellular Vesicle Subpopulations. Organic Biomol Chem (2015) 13:9775–82. doi: 10.1039/c5ob01451d 26264754

[23] Han L Lam E Sun Y. Extracellular Vesicles in the Tumor Microenvironment: Old Stories, But New Tales. Mol Cancer (2019) 18:59. doi: 10.1186/s12943-019-0980-8 30925927 PMC6441234

[24] Maacha S Bhat A Jimenez L Raza A Haris M Uddin S. Extracellular Vesicles-Mediated Intercellular Communication: Roles in the Tumor Microenvironment and Anti-Cancer Drug Resistance. Mol Cancer (2019) 18:55. doi: 10.1186/s12943-019-0965-7 30925923 PMC6441157

[25] Lötvall J Hill A Hochberg F Buzás E Di Vizio D Gardiner C. Minimal Experimental Requirements for Definition of Extracellular Vesicles and Their Functions: A Position Statement From the International Society for Extracellular Vesicles. J Extracell Vesicles (2014) 3:26913. doi: 10.3402/jev.v3.26913 25536934 PMC4275645

[26] Neumann M Bender S Krahn T Schlange T. ctDNA and CTCs in Liquid Biopsy - Current Status and Where We Need to Progress. Comput Struct Biotechnol J (2018) 16:190–5. doi: 10.1016/j.csbj.2018.05.002 PMC6024152 29977481

[27] Zhao L Sun L Chu X. Chemiluminescence Immunoassay. TrAC Trends Anal Chem (2009) 28:404–15. doi: 10.1016/j.trac.2008.12.006

[28] Akhavan-Tafti H Binger DG Blackwood JJ Chen Y Creager RS de Silva R. A Homogeneous Chemiluminescent Immunoassay Method. J Am Chem Soc (2013) 135:4191–4. doi: 10.1021/ja312039k 23477541

[29] Campos-Silva C Suárez H Jara-Acevedo R Linares-Espinós E Martinez-Piñeiro L Yáñez-Mó M. High Sensitivity Detection of Extracellular Vesicles Immune-Captured From Urine by Conventional Flow Cytometry. Sci Rep (2019) 9:2042. doi: 10.1038/s41598-019-38516-8 30765839 PMC6376115

[30] Went P Lugli A Meier S Bundi M Mirlacher M Sauter G. Frequent EpCam Protein Expression in Human Carcinomas. Hum Pathol (2004) 35:122–8. doi: 10.1016/j.humpath.2003.08.026 14745734

[31] Sankpal NV Fleming TP Sharma PK Wiedner HJ Gillanders WE. A Double-Negative Feedback Loop Between EpCAM and ERK Contributes to the Regulation of Epithelial-Mesenchymal Transition in Cancer. Oncogene (2017) 36:3706–17. doi: 10.1038/onc.2016.504 PMC5571977 28192403

[32] Gao J Yan Q Wang J Liu S Yang X. Epithelial-To-Mesenchymal Transition Induced by TGF-Beta1 Is Mediated by AP1-Dependent EpCAM Expression in MCF-7 Cells. J Cell Physiol (2015) 230:775–82. doi: 10.1002/jcp.24802 25205054

[33] Ye X Tam W Shibue T Kaygusuz Y Reinhardt F Ng Eaton E. Distinct EMT Programs Control Normal Mammary Stem Cells and Tumour-Initiating Cells. Nature (2015) 525:256–60. doi: 10.1038/nature14897 PMC4764075 26331542

[34] Kalaany N Sabatini D. Tumours With PI3K Activation Are Resistant to Dietary Restriction. Nature (2009) 458:725–31. doi: 10.1038/nature07782 PMC2692085 19279572

[35] Fraga A Ribeiro R Príncipe P Lopes C Medeiros R. Hypoxia and Prostate Cancer Aggressiveness: A Tale With Many Endings. Clin Genitourin Cancer (2015) 13:295–301. doi: 10.1016/j.clgc.2015.03.006 26007708

[36] Li X Lao Y Zhang H Wang X Tan H Lin Z. The Natural Compound Guttiferone F Sensitizes Prostate Cancer to Starvation Induced Apoptosis *via* Calcium and JNK Elevation. BMC Cancer (2015) 15:254. doi: 10.1186/s12885-015-1292-z 25885018 PMC4394563

[37] Panigrahi GK Praharaj PP Peak TC Long J Singh R Rhim JS. Hypoxia-Induced Exosome Secretion Promotes Survival of African-American and Caucasian Prostate Cancer Cells. Sci Rep (2018) 8:3853. doi: 10.1038/s41598-018-22068-4 29497081 PMC5832762

[38] Umezu T Tadokoro H Azuma K Yoshizawa S Ohyashiki K Ohyashiki J. Exosomal miR-135b Shed From Hypoxic Multiple Myeloma Cells Enhances Angiogenesis by Targeting Factor-Inhibiting HIF-1. Blood (2014) 124:3748–57. doi: 10.1182/blood-2014-05-576116 PMC4263983 25320245

[39] Ramteke A Ting H Agarwal C Mateen S Somasagara R Hussain A. Exosomes Secreted Under Hypoxia Enhance Invasiveness and Stemness of Prostate Cancer Cells by Targeting Adherens Junction Molecules. Mol Carcinog (2015) 54:554–65. doi: 10.1002/mc.22124 PMC4706761 24347249

