# Supplementary material for: A Multivariate Diagnostic Model Based on Urinary EpCAM-CD9-Positive Extracellular Vesicles for Prostate Cancer Diagnosis
Source: Front Oncol. 2021 Nov 24;11:777684. doi: 10.3389/fonc.2021.777684 (PMC8652292; doi:10.3389/fonc.2021.777684)
Supplement: Supplementary file 1 [file DataSheet_1.docx]

**Supplemental materials**

**Supplementary Figure 1. Optimize the critical parameters based on chemiluminescent immunoassay of PC3 EVs.** Optimize the reaction conditions and parameters of the EV detection assay, including the concentration of streptavidin-labeled magnetic beads (A), biotin-labeled anti-EpCAM antibodies (B), ACE-labeled anti-CD9 antibodies (C) and reaction time (D). RCU, relative chemiluminescent unit; EVs, extracellular vesicles; EpCAM, epithelial cell adhesion molecule; ACE, acridinium ester.


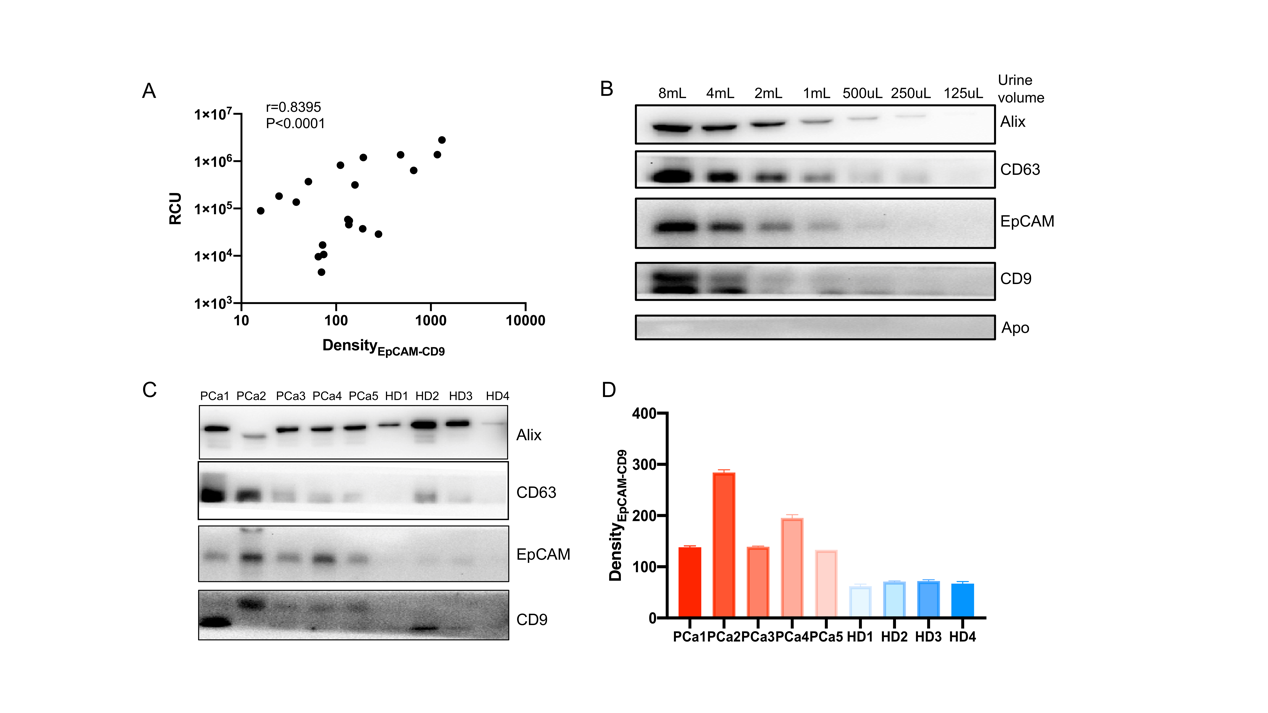


**Supplementary Figure 2. The uEV_EpCAM-CD9_ of PCa and HD detected by WB.** (A) The correction of Density_EpCAM-CD9_ and chemiluminescent signals. (B) The protein profile from different urine volume revealed the presence of EpCAM and CD9 positive EVs in PCa. (C-D) The uEV_EpCAM-CD9_ from 9 randomly selected donors including 5 PCa and 4 HD were assayed by WB. RCU, relative chemiluminescent unit; uEV_EpCAM-CD9_, urinary EpCAM-CD9-positive extracellular vesicles; PCa, prostate cancer; HD, healthy donor; WB, western blot; Density_EpCAM-CD9_, EpCAM-CD9 protein density.

**Table S1. Clinical characteristics of the men with PCa patients and without PCa**

| Variable | Men with PCa(n=112) | Men without PCa(n=81) | P value |
| --- | --- | --- | --- |
|  | median(IQR) or n(%) | median(IQR) or n(%) |  |
| Age(years) | 72(65-76) | 64(53-72) | <0.0001 |
| Smoking |  |  |  |
| Yes | 50（44.6） | 20（24.7） | 0.005 |
| No | 62（55.4） | 61（75.3） |  |
| Drinking |  |  |  |
| Yes | 47（42.0） | 20（24.7） | 0.013 |
| No | 65（58.0） | 61（75.3） |  |
| Family history |  |  |  |
| Yes | 11（9.8） | 3（3.7) | 0.107 |
| No | 101（0.9） | 78(96.3) |  |
| BMI(kg/m² ) | 23.53(21.84-25.54) | 22.49(21.50-24.55) | 0.016 |
| Gleason score |  |  | <0.0001 |
| 6 | 17 （15.2） | NA |  |
| 7 | 49 （43.8） | NA |  |
| 8 | 21 （18.8） | NA |  |
| 9-10 | 25 （22.3） | NA |  |
| CEA(ng/mL) | 2.6(1.8-3.5) | 2.0(1.5-3.0) | 0.007 |
| AFP(ng/mL) | 2.6(1.8-3.3) | 2.5(1.8-3.5) | 0.428 |
| CA125(U/mL) | 10.5(7.9-13.8) | 11.4(6.7-13.5) | 0.980 |
| CA199(U/mL) | 6.7(4.2-11.4) | 7.0(3.8-11.2) | 0.615 |
| EpCAM-CD9-positive EV concentration(n.u) | 1.46(0.86-2.66) | 0.55(0.22-0.84) | <0.0001 |
| PSA(ng/mL) | 11.5055(8.4370-28.9283) | 2.6780(0.9930-6.1465) | <0.0001 |
| fPSA(ng/mL) | 1.6712(1.0644-3.1130) | 0.7140(0.2855-1.4400) | <0.0001 |
| f/T PSA | 0.13(0.08-0.20) | 0.22(0.18-0.31) | <0.0001 |
| PV(cm³) | 61.50(45.76-94.09) | 66.38(24.00-107.39) | 0.543 |
| PSAD(ng/mL²) | 0.23(0.11-0.44) | 0.04(0.02-0.07) | <0.0001 |

PCa, prostate cancer; BMI, body mass index; EV, extracellular vesicle; PSA, prostate-specific antigen; fPSA, free prostate-specific antigen; f/T PSA, free/total prostate-specific antigen; PV, prostate volume; PSAD, prostate-specific antigen density; IQR, interquartile range; NA, not applicable.

| EVs | For EVs from the cell line supernatant | | |  | For EVs from urinary samples | | |
| --- | --- | --- | --- | --- | --- | --- | --- |
|  | Recovery (%) | Intra-CV (%) | Inter-CV (%) |  | Recovery (%) | Intra-CV (%) | Inter-CV (%) |
| Low Concentration | 85.45 | 9.72 | 18.06 |  | 92.36 | 4.28 | 10.61 |
| Medium Concentration | 95.45 | 8.90 | 13.60 |  | 95.40 | 9.17 | 11.15 |
| High Concentration | 101.65 | 7.65 | 11.44 |  | 108.31 | 2.11 | 10.56 |

**Table S2. Experimental data of the recovery test and repeatability test.**

EVs, extracellular vesicles; intra-CV, intra-assay coefficient of variation; inter-CV, inter-assay coefficient of variation.

**Table S3. The multivariate diagnostic models assessed by their AUC in ROC curve analysis**

| Model | AUC | 95% CI | Specificity | Sensitivity | P value |
| --- | --- | --- | --- | --- | --- |
| CF + uEV + PV + PSA + f/T PSA + PSAD + fPSA | 0.963 | 0.9333-0.9927 | 0.936 | 0.899 | <0.001 |
| CF + uEV + PV + PSA + f/T PSA + PSAD | 0.962 | 0.9314-0.9933 | 0.979 | 0.855 | <0.001 |
| CF + uEV + PV + PSA + PSAD | 0.961 | 0.9288-0.9929 | 0.979 | 0.855 | <0.001 |
| CF + uEV + PV + PSA + f/T PSA | 0.954 | 0.9202-0.9879 | 0.851 | 0.942 | <0.001 |
| CF + uEV + PV + PSA | 0.952 | 0.9173-0.9871 | 0.851 | 0.942 | <0.001 |
| CF + uEV + PV + f/T PSA + PSAD | 0.946 | 0.9079-0.9836 | 0.979 | 0.797 | <0.001 |
| CF + uEV + PSA + f/T PSA + PSAD | 0.944 | 0.9074-0.9815 | 0.766 | 0.971 | <0.001 |
| CF + uEV + PSA + PSAD | 0.944 | 0.9070-0.9814 | 0.787 | 0.957 | <0.001 |
| CF + uEV + PV + PSAD | 0.936 | 0.8945-0.9785 | 0.872 | 0.870 | <0.001 |
| CF + uEV + PSA + f/T PSA | 0.93 | 0.8858-0.9742 | 0.766 | 0.971 | <0.001 |
| CF + uEV + f/T PSA + PSAD | 0.929 | 0.8741-0.9522 | 0.979 | 0.768 | <0.001 |
| CF + PV + PSA + f/T PSA | 0.928 | 0.8806-0.9744 | 0.894 | 0.870 | <0.001 |
| CF + PV + PSA + f/T PSA + PSAD | 0.926 | 0.8786-0.9728 | 0.809 | 0.928 | <0.001 |
| CF + PV + PSA + PSAD | 0.925 | 0.8792-0.9703 | 0.851 | 0.841 | <0.001 |
| CF + uEV + PV + f/T PSA | 0.908 | 0.8552-0.9604 | 0.894 | 0.783 | <0.001 |
| CF + PSA + f/T PSA + PSAD | 0.908 | 0.8681-0.9472 | 0.809 | 0.870 | <0.001 |
| CF + PV + f/T PSA + PSAD | 0.896 | 0.8378-0.9544 | 0.809 | 0.884 | <0.001 |

AUC, area under the curve; ROC, receiver operating characteristic; CF: Age + Smoking + Drinking + Family history + BMI; uEV: Log urinary EpCAM-CD9-positive extracellular vesicles concentration (n.u); PV: prostate volume; PSA: prostate-specific antigen; f/T PSA: free/total prostate-specific antigen; PSAD: prostate-specific antigen density; CI, confidence interval.
